# Novel Calculation Method for the Shear Capacity of a UHPC Beam with and without Web Reinforcement

**DOI:** 10.3390/ma16216915

**Published:** 2023-10-27

**Authors:** Chuansong Gao, Hui Jiang, Gaozhan Zhang, Liang Chen, Yuqing Hu

**Affiliations:** 1 BIM Engineering Center of Anhui Province, Anhui Jianzhu University, Hefei 230601, China; 220211248@seu.edu.cn; 2 Department of Civil Engineering, Southeast University, Nanjing 210096, China; 3 College of Civil Engineering, Xuzhou University of Technology, Xuzhou 221111, China; jianghui@xzit.edu.cn; 4 Advanced Building Materials Key Laboratory of Anhui Province, Anhui Jianzhu University, Hefei 230022, China; gaozhanzhang@ahjzu.edu.cn; 5 College of Civil Engineering, Hefei University of Technology, Hefei 230009, China; popecl@hfut.edu.cn; 6 State Key Laboratory of Safety, Durability and Healthy Operation of Long Span Bridges, Nanning 210037, China

**Keywords:** UHPC beam, cracking mode, shear capacity, compression zone, fiber bridging effect, failure mechanism

## Abstract

To accurately predict the shear-bearing capacity of UHPC beams, it is crucial to quantify the shear contribution of the fiber bridging effect and UHPC compression zone. Nevertheless, it should be noted that the shear contribution of UHPC in the compression zone is not fully considered in most existing calculation methods, and the probability distribution of fibers within the matrix is also not taken into full account, which reduces the calculation accuracy of the shear bearing capacity of UHPC beams. In this paper, a UHPC beam shear test database containing 247 samples was created, and the influencing factors on the shear capacity of UHPC beams, such as the shear span ratio, the web reinforcement ratio, and the volume fraction of steel fiber, were analyzed. It was found that the ratio of cracking load to ultimate load ranges from 0.2 to 0.6, and the failure in the compression zone of UHPC beams can be divided into diagonal tension failure and shear compression failure. Based on the failure mechanism of the compression zone, considering the contribution of fiber micro tensile strength, a formula for calculating the shear-bearing capacity of UHPC beams with and without web reinforcement was proposed. Verified by experimental data, the proposed formula accurately predicts the shear-bearing capacity of UHPC beams. In comparison with other shear capacity formulas in current design codes, the proposed formula in this paper provides a higher prediction accuracy.

## 1. Introduction

Due to the optimized gradation of granular constituents and the addition of steel fibers, ultra-high performance concrete (UHPC) is considered to be one of the most innovative cement-based materials of the past thirty years with an ultra-high compressive and high tensile strength and excellent durability [1]. Currently, the number of implementations for UHPC in beam structures is increasing rapidly around the world, resulting in many new problems for their shear design [2,3].

Regarding traditional concrete beams, shear-strength models, such as the truss model, strut-and-tie model (STM), and modified compression field model (MCFT) etc., were proposed to predict their shear strength [4,5,6,7,8]. The shear strength of UHPC is closely related to the mechanical behavior in tension and compression conditions of UHPC. De Maio et al. [9,10] conducted a failure analysis of ultra-high performance fiber-reinforced concrete structures enhanced with nanomaterials by using a diffuse cohesive interface approach and developed an integrated numerical model relying on a diffuse cohesive interface approach, which probably accurately predicts multiple crack initiation and propagation phenomena and preserves the discrete nature of fracture processes. In addition, due to the addition of fibers, UHPC presents strain hardening and post-cracking behavior, and its tensile strength and ductility are also improved in comparison with traditional concrete. Tian et al. [11] conducted an investigation on the eccentric compressive behavior of fiber-reinforced polymer (FRP) confined UHPC and found significant strain hardening, where UHPC was treated as a two-phase material and simulated with UHPC matrix and explicitly modeled steel fibers. The above resulted in traditional shear design methods for concrete beams not being applicable to UHPC beams.

At present, the influences of the fiber content [12,13], fiber direction [14,15], prestressing level [16,17], and the shear span ratio [18,19,20] on the shear performance of UHPC beams were investigated, and some shear calculation methods for UHPC beams were also proposed. In these calculation methods, the statistical analysis method [21,22] is convenient to use, but it is limited by statistical samples and cannot conform completely to the shear mechanism of UHPC beams. The plastic theory method [12,23] typically introduces plastic shear coefficients, but these coefficients in formulas have no clear physical meanings. Compared with the above two methods, the ultimate equilibrium method [24,25] can analyze the stress states of each component from a microscopic perspective. Nevertheless, it should be noted that the probability distribution of fibers in the matrix has not been reasonably considered in the current ultimate equilibrium method. In addition, the modified pressure field theory [26,27] effectively analyzes the UHPC constitutive relationship, which is useful for finite-element analysis, but it is not convenient for design purposes.

Through analysis of the four above calculation methods, it can be found that the accurate quantification of the fiber bridging effect is crucial for achieving accurate prediction of the shear-bearing capacity of UHPC beams. Currently, two approaches, treating the fiber and matrix as homogenous materials or as separate materials, are used to quantify the fiber bridging effect. With the first approach, the bridging contribution of fibers is obtained by multiplying the tensile strength of UHPC with the surface area of diagonal cracks. This calculation method of the fiber bridge effect has been included in some specifications, such as Design Guidelines for Ductal Prestressed Concrete Beams [28] and Ultra-High Performance Fibre Reinforced Concretes-Interim Recommendations [29]. However, it should be noted that they ignore the orientation and depth of fibers and cannot accurately reflect the true shear resistance mechanism of fibers. The latter approach, which treats the fiber and matrix as separate materials, can accurately reflect the contribution of fibers to shear resistance in UHPC beams [24,25,26,27,30], and it considers the depth and orientation of fibers.

However, the influences of the probability distribution of fibers and shear span ratio on the shear capacity of UHPC beams are not taken into full account in most existing calculation methods. In addition, due to the superior compressive performance of UHPC compared to normal concrete (NC), the shear contribution of UHPC in the compression zone is significantly increased compared to that of NC. However, the contribution of the ultra-high compressive strength of UHPC in the compression zone is usually not fully considered in most existing calculation methods, which reduces the calculation accuracy.

In order to accurately predict the shear bearing capacity of UHPC beams, it is necessary to propose a UHPC beam shear strength calculation formula that precisely quantifies the shear contributions of fibers and compressive strength of UHPC in the compression zone. Therefore, in this paper, a database was established to analyze the influence of various factors, such as the shear-span ratio, reinforcement ratio, web reinforcement ratio, fiber volume fraction, and the UHPC compressive strength, on the shear capacity of UHPC beams. The failure mechanism and cracking mode of UHPC beams were revealed. Based on the failure modes and shear cracking mode, the shear contributions provided by the steel fibers and the UHPC compression zone were accurately quantified using composite mechanics, and then a formula for calculating the shear capacity of UHPC beams with and without web reinforcement was proposed. The accuracy of calculation formulas in the current UHPC design codes was evaluated, and the accuracy of the proposed formula in this paper was verified.

## 2. Analysis of Influencing Factors on the Shear Capacity Based on Database

### 2.1. Database Construction

**Figure 1:**
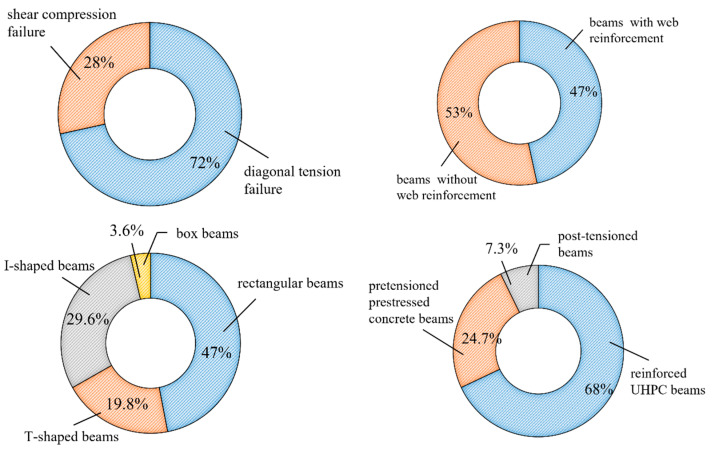
Shear database statistics.

The shear test results of 247 UHPC beams from domestic and international literature [2,3,12,15,16,17,18,19,25,31,32,33,34,35,36,37,38,39,40,41,42,43,44,45,46,47] were collected to establish the shear database of UHPC beams. In this database (Figure 1), 53% of beams have web reinforcement, and 47% of beams have no web reinforcement. In terms of types of reinforcement, the reinforced UHPC beams account for 68%, and prestressed UHPC beams account for 32%. Note that most of the prestressed UHPC beams are pre-tensioned prestressed beams. In terms of beam section, rectangular section beams account for 47%, T and I beams account for 49.4%, and box section beams account for only 3.6%.

**Figure 2:**
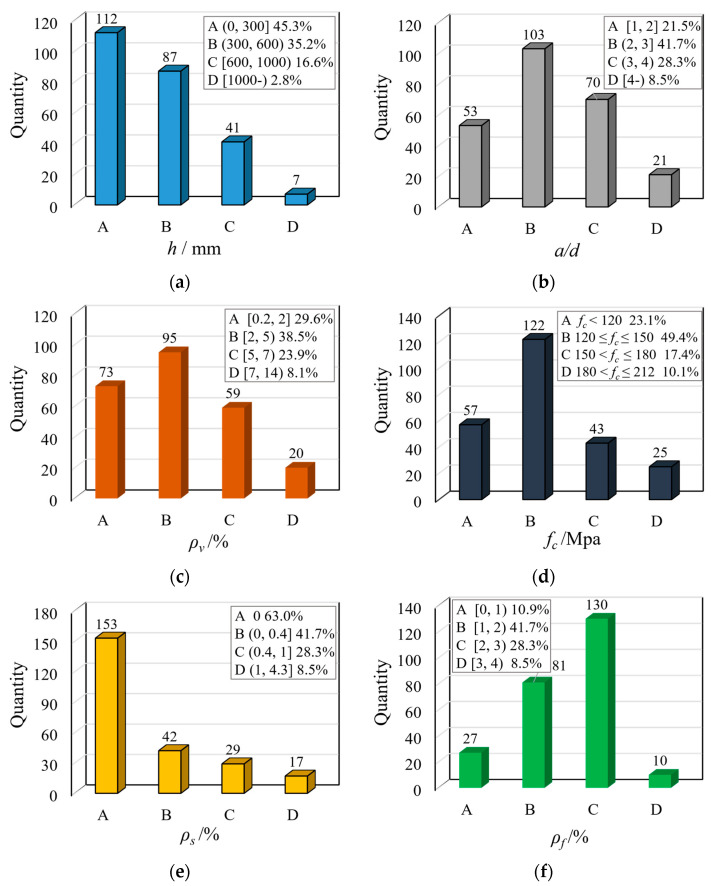
Parameter distribution of the shear database: (**a**) beam depth (*h*); (**b**) shear span ratio (*a/d*); (**c**) longitudinal reinforcement ratio (*ρ_v_*); (**d**) UHPC compressive strength (*f_c_*); (**e**) web reinforcement ratio (*ρ_s_*); and (**f**) volume fraction of steel fiber (*ρ_f_*).

The parameter distribution in the database, which influences the shear performance of UHPC beams, is shown in Figure 2 as follows: 140 mm≤h≤1092 mm, 1.0≤a/d≤4.5, $0.2\%\leq \rho_{v}\leq 13.6\%$, $73.8\;MPa\leq f_{c}\leq 212\;MPa$, $0\%\leq \rho_{s}\leq 4.3\%$, $0\%\leq \rho_{f}\leq 5.0\%$; where h is the beam depth, a/d is the shear span ratio, $\rho_{v}$ is the longitudinal reinforcement ratio, $f_{c}$ is the compressive strength of UHPC, $\rho_{s}$ is the web reinforcement ratio, and $\rho_{f}$ is the volume fraction of steel fiber. The experimental parameters in this database cover a wide range and can better reveal the shear performance of UHPC beams. Note that the UHPC beam, of which UHPC material has no fibers within a lower compressive strength of 73.8 MPa, is also included in the database to systematically evaluate the shear strength of UHPC beams.

### 2.2. Analysis of Influencing Factors on the Shear Capacity

**Figure 3:**
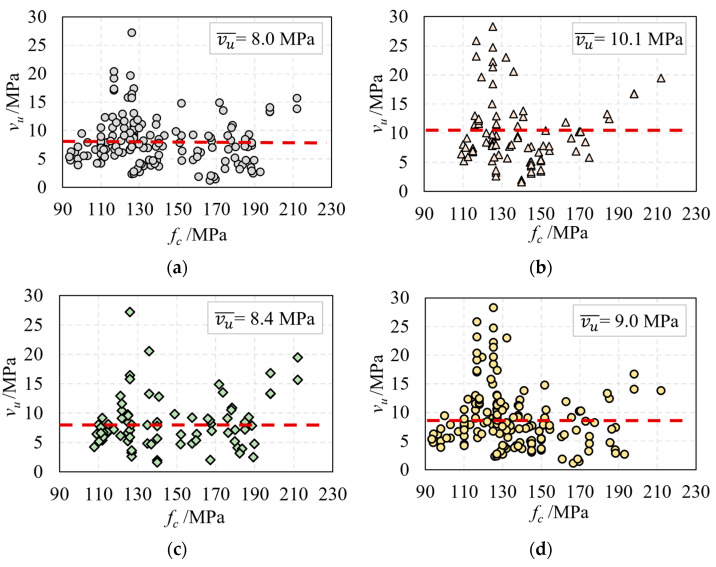
The relationship of $f_{c}$ and $v_{u}$: (**a**) the beam with web reinforcement; (**b**) the beam without web reinforcement; (**c**) the prestressed beam; and (**d**) the non-prestressed beam.

**Figure 4:**
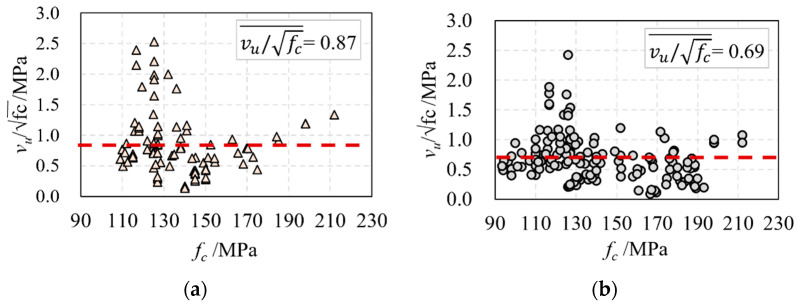
The relationship of $v_{u}/\sqrt{f_{c}}$ and $f_{c}$: (**a**) the beam with web reinforcement and (**b**) the beam without web reinforcement.

**Figure 5:**
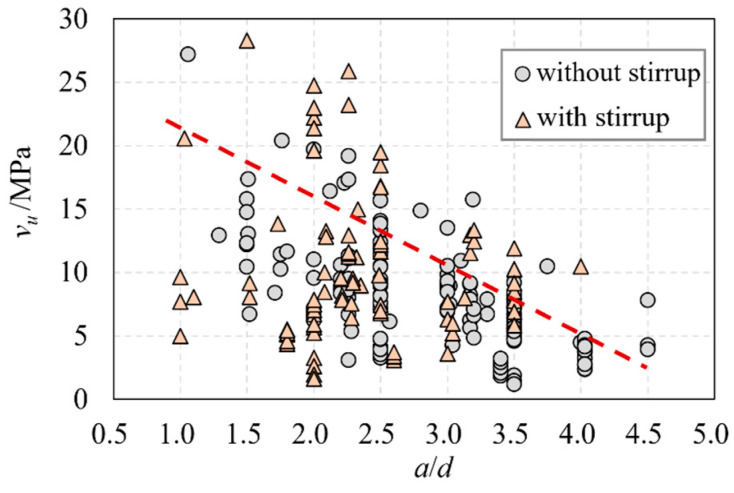
The relationship of a/d and $v_{u}$.

**Figure 6:**
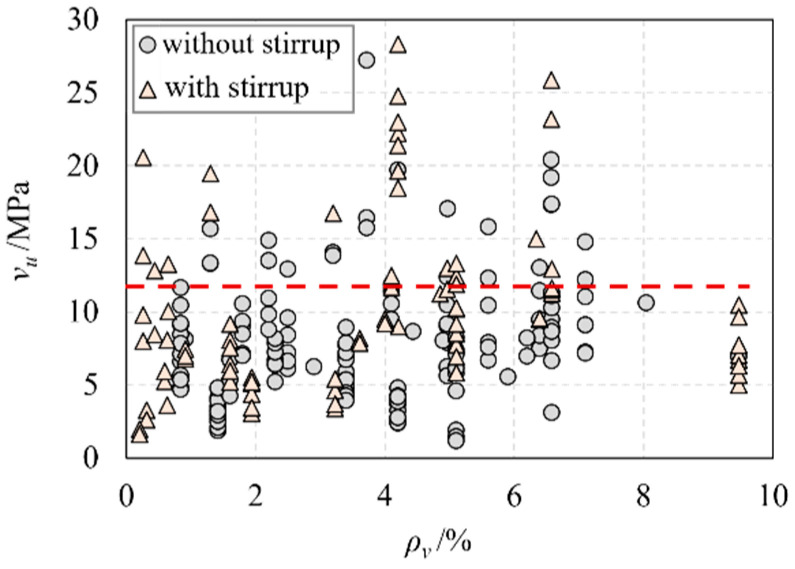
The relationship of $\rho_{v}$ and $v_{u}$.

**Figure 7:**
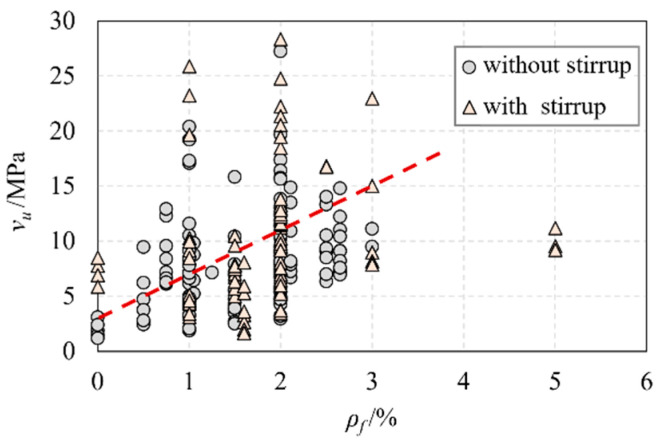
The relationship of $\rho_{f}$ and $v_{u}$.

To facilitate the analysis of the influence of various parameters on the shear performance of UHPC beams, the nominal shear capacity ($v_{u}=V_{u}/bh$) was used as a benchmark. Figure 3 illustrates the relationship between UHPC compressive strength and nominal shear capacity. It was found that the influence of the UHPC compressive strength on the nominal shear capacity is not significant if the shear span ratio is not considered, as shown in Figure 3a,b. The red line represents the average value in Figure 3, Figure 4, Figure 5, Figure 6 and Figure 7.

According to [48], the direct tensile strength of UHPC is $f_{dt}/\sqrt{f_{c}}=0.56$ under the natural curing condition and $f_{dt}/\sqrt{f_{c}}=0.65$ under steam curing conditions. As shown in Figure 4, the average $v_{u}/\sqrt{f_{c}}$ of reinforced UHPC beams is 0.69, and the average $v_{u}/\sqrt{f_{c}}$ of prestressed UHPC beams is 0.87, indicating that prestress can improve the shear capacity of UHPC beams. Comparing the two values of $v_{u}/\sqrt{f_{c}}$ with the value of $f_{dt}/\sqrt{f_{c}}$, it is found that the shear strength of UHPC beams is greater than that of the tensile strength.

Influenced by the failure mode, the $v_{u}$ of UHPC beams decreases with the increase of the shear span ratio (a/d), as shown in Figure 5. However, it is worth noting that the influence of the shear span ratio on the shear-bearing capacity was not taken into full account in the calculation formulas used in current UHPC structural codes, which is obviously unreasonable.

Figure 6 shows the relationship between the nominal shear stress ($v_{u}$) and the longitudinal reinforcement ratio ($\rho_{v}$). Interestingly, the longitudinal reinforcement ratio has no significant influence on the nominal shear stress. Figure 7 demonstrates the relationship between the nominal shear stress ($v_{u}$) and the fiber volume fraction ($\rho_{f}$). It was found that increasing the fiber volume fraction enhances the shear performance of UHPC beams if the fiber volume fraction exists in a certain range. However, the shear capacity of UHPC beams will not continue to increase if the fiber volume fraction exceeds a valid range.

**Figure 8:**
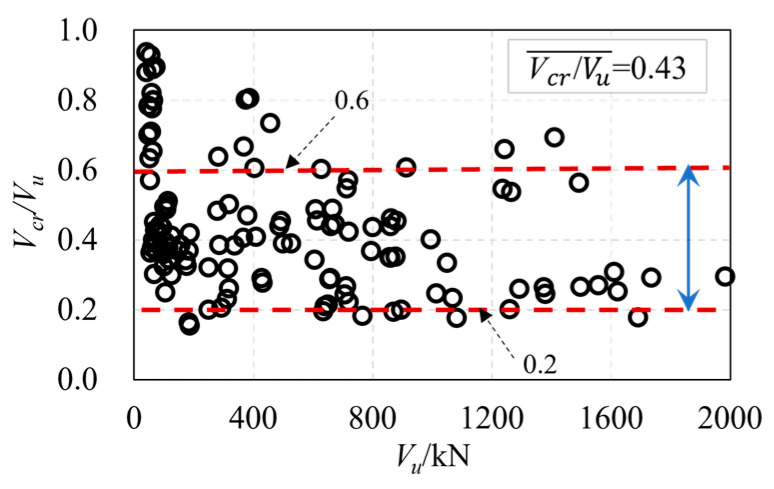
The relationship of $V_{cr}/V_{u}$ and $V_{u}$.

**Figure 9:**
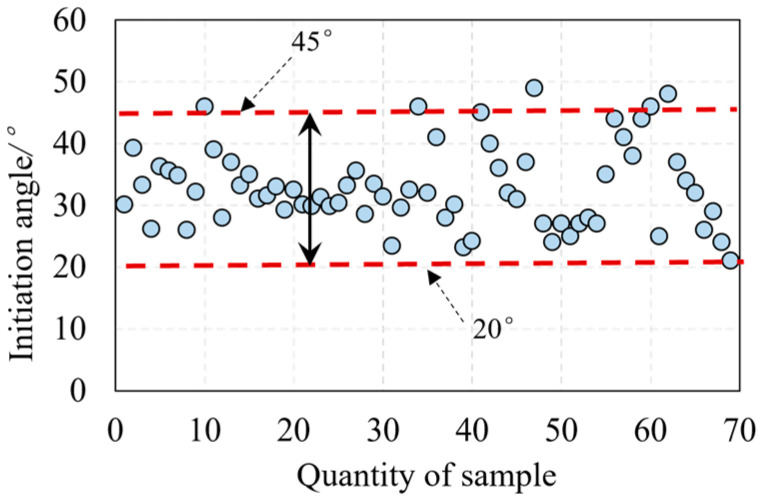
Cracking angle of the main oblique crack.

The relationship between cracking and the ultimate strength of UHPC beams obtained from test results is shown in Figure 8. It was found that the ratio of cracking load to ultimate load ranges from 0.2 to 0.6, with a mean average of 0.43. This result indicates that the shear cracking of UHPC beams generates a low load level. Thus, the shear cracking load level of UHPC beams at serviceability limit states (SLS) should be taken into account in the design to avoid the existence of shear cracks at SLS. On the other hand, the results can conclude that the process from cracking to shear failure is long, indicating a better ductility of UHPC beams. Figure 9 shows that the critical inclined angles of the oblique main crack range from 20° to 45°, with the majority of angles concentrated around 30°.

## 3. The Calculation and Analysis of Shear Bearing Capacity of UHPC Beams

**Figure 10:**
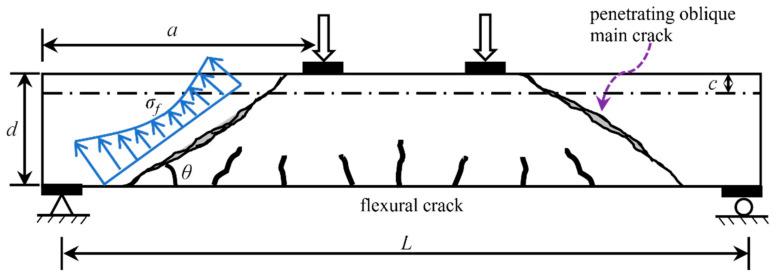
Cracking pattern of diagonal tension failure in compression zone (mode 1).

**Figure 11:**
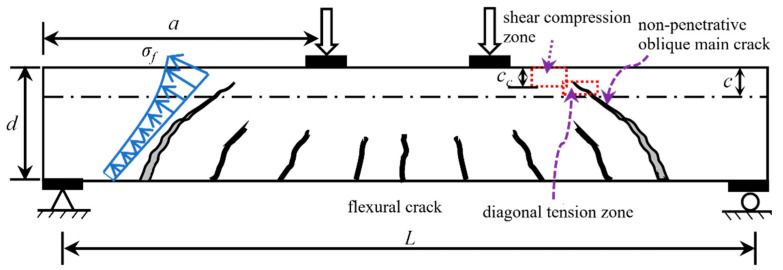
Cracking pattern of shear compression failure in compression zone (mode 2).

The shear failure modes of UHPC beams are generally similar to those of reinforced concrete (RC) beams. However, the cracking patterns of UHPC beams differ from those of RC beams. As shown in Figure 10, the UHPC beam with a low-ratio web reinforcement or without web reinforcement exhibits a major diagonal crack taking the form of a ‘narrow-wide-narrow’ and manifesting as the diagonal tension failure. If the UHPC beam has a thick web or high-ratio web reinforcement, it exhibits a major diagonal crack in the form of a ‘wide bottom and narrow top’, as shown in Figure 11. Due to the inhibition of web reinforcement, the main diagonal crack will not extend beyond a certain height upon developing upward from the bottom of the beam. The difference in the cracking patterns and failure modes in the compression zone of these two types of beams is apparent.

**Figure 12:**
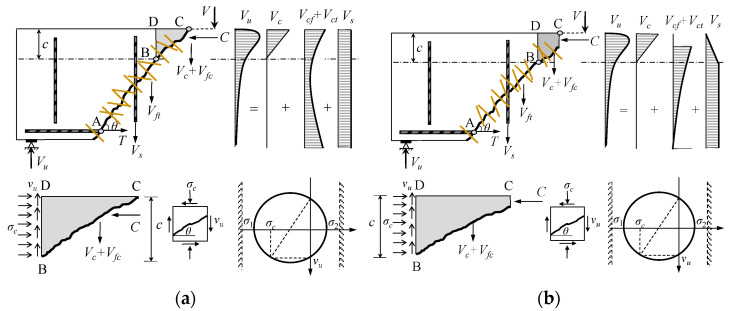
Calculation model based on the failure mode of compression zone: (**a**) diagonal tensile failure (model 1) and (**b**) shear compression failure (model 2).

When the compression zone of the UHPC beam presents diagonal tension failure, the shear diagonal crack penetrates the compression zone, eliminating the shear compression effect in the compression zone. Thus, the shear contribution of the compression zone is provided by the compressive and tensile strength of UHPC in the compression zone. Figure 12a provides a schematic representation, where $V_{c}$, $V_{fc}+V_{ft}$, and $V_{s}$ are the shear contributions provided by the UHPC’s compressive strength, the fiber bridging effect in the inclined section, and the web reinforcement tensile strength, respectively.

If the compression zone of the UHPC beam experiences shear compression failure, the shear diagonal crack does not penetrate the compression zone. The compression zone is composed of the diagonal tension zone and the shear compression zone. The shear contribution is divided into two parts as follows: the contribution provided by UHPC’s tensile strength and the contribution provided by the shearing strength of UHPC in the compression zone, as illustrated in Figure 12b.

According to the calculation model, the shear capacity of UHPC beams with web reinforcement is comprised of three elements: the compressive strength of UHPC ($V_{c}$), the fiber bridging effect in the inclined section ($V_{fc}+V_{ft}$), and the tensile strength of web reinforcement ($V_{s}$). Regarding UHPC beams without web reinforcement, their shear capacity is only related to the compressive strength of UHPC ($V_{c}$) and the fiber bridging effect in the inclined section ($V_{fc}+V_{ft}$). Both calculation models in Figure 12 consider the shear contribution of web reinforcement and fibers, while the shear contribution provided by UHPC differs due to the different failure modes in the compression zone. Furthermore, the shear contribution of fibers in these two models differs because of the varying cracking characteristics of shear diagonal cracks.

### 3.1. Quantification of Shear Contributions in the Compression and Tension Zones

#### 3.1.1. Shear Contributions in the Compression Zone

According to the Rankine failure criterion, the shear strength of UHPC in the compression zone is mainly controlled by its tensile strength when the diagonal tensile failure occurs. If shear compression failure occurs, the failure of the shear compression zone follows the principal compressive stress failure criterion, which means the shear strength of UHPC in the compression zone is mainly determined by its compressive strength. When diagonal tensile failure and shear compression failure occur in the compression zone, the stress state can respectively be expressed as:

(1) $$\;\sigma_{1}=-\frac{\sigma_{y}}{2}+\sqrt{{\left(\frac{\sigma_{y}}{2}\right)}^{2}+\tau_{xy}^{2}}=f_{t}$$

(2) $$\;\sigma_{2}=-\frac{\sigma_{y}}{2}+\sqrt{{\left(\frac{\sigma_{y}}{2}\right)}^{2}+\tau_{xy}^{2}}=f_{c}$$

where $\sigma_{1}$ and $\sigma_{2}$ is the principal stress, $\sigma_{y}$ is normal stress along beam height, $\tau_{xy}$ is the shear stress, $f_{c}$ is compressive strength of UHPC, and $f_{t}$ is tensive strength of UHPC.

Equation (1) can be further simplified as:

(3) $$\tau_{xy}^{2}=f_{t}^{2}+f_{t}\sigma_{y}$$

**Figure 13:**
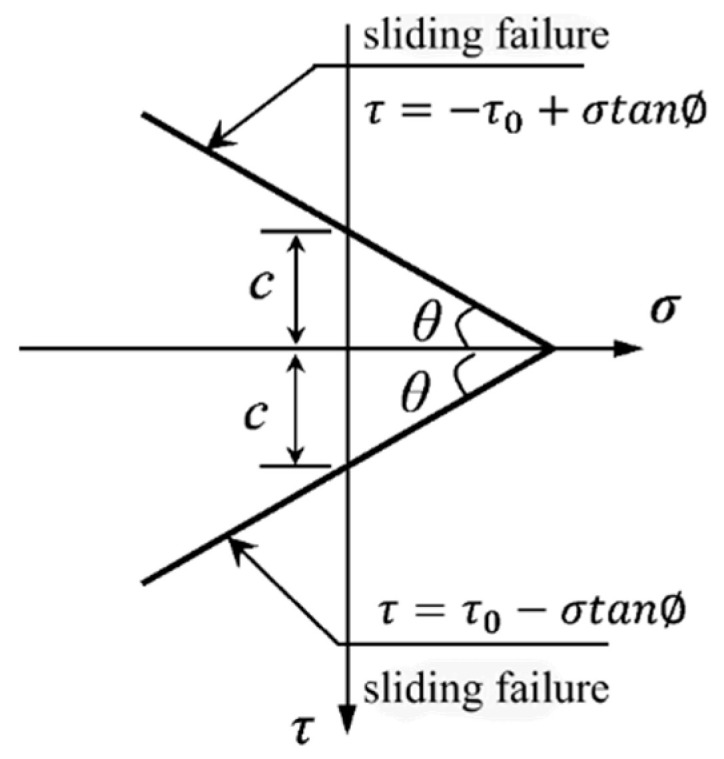
Modified Mohr-Coulomb failure criterion.

The modified Mohr-Coulomb (M-C) failure criteria (Figure 13) can be expressed as:

(4) $$\left|\tau \right|=\tau_{0}-\sigma_{y}\tan\phi$$

where $\left|\tau \right|$ is ultimate shearing strength, $\tau_{0}$ is cohesion, and ϕ is the friction angle.

Then, Equation (5) is obtained by combining Equations (2) and (3):

(5) $${\left(\tau_{0}-\sigma_{y}tan\phi \right)}^{2}=f_{t}^{2}+f_{t}+f_{t}\sigma_{y}$$

And Equation (5) can be further written as:

(6) $${\left(\sigma_{y}\tan\phi \right)}^{2}-\left(2\tau_{0}\tan\phi +f_{t}\right)\sigma_{y}+\left(\tau_{0}^{2}-f_{t}^{2}\right)=0$$

The solution of $\sigma_{y}$ is obtained by placing $\tau_{0}=\sqrt{{f_{c}f}_{t}/2}$ [49] in Equation (6). Substituting it into equation Equation (6) can be solved as:

(7) $$\sigma_{y}=3.7f_{t}$$

When the diagonal tensile failure occurs in the compression zone, the shear contribution provided by the compressive strength of UHPC can be expressed as:

(8) $$V_{c}=b\int_{0}^{c}\tau_{xy}dy=b\int_{0}^{c}\sqrt{f_{t}^{2}+f_{t}\sigma_{y}}dy$$

where b is the thickness of web, c is the height of compression zone, and y is the height of the coordinate.

If UHPC beams exhibit tensile failure in the compression zone, UHPC is considered to be in an elastic state owing to its ultra-high compressive strength. Thus, the normal stress along the height of the compression zone is assumed as a linear distribution, and the equation can be written as:

(9) $$\;\sigma_{y}=\frac{3.7f_{t}}{c}y$$

Then, the shear contribution provided by the compressive strength of UHPC in the diagonal tensile zone is obtained as follows:

(10) $$\;V_{c}=b\int_{0}^{c}\sqrt{f_{t}^{2}+f_{t}\sigma_{y}}dy=b\int_{0}^{c}\sqrt{f_{t}^{2}\left(1+\frac{3.73}{c}y\right)}dy=1.66f_{t}bc$$

According to Leutbeche and Rebling [50], $f_{t}$ represents residual tensile strength here, and $f_{t}=0.3\sigma_{t}$. Equation (10) is further expressed as:

(11) $$V_{c}=0.5\sigma_{t}bc$$

When the compression zone experiences shear compression failure, the compressive strength of UHPC controls its failure. Equation (2) can be expressed as:

(12) $$\tau_{xy}^{2}=f_{c}^{2}-f_{c}\sigma_{y}$$

**Figure 14:**
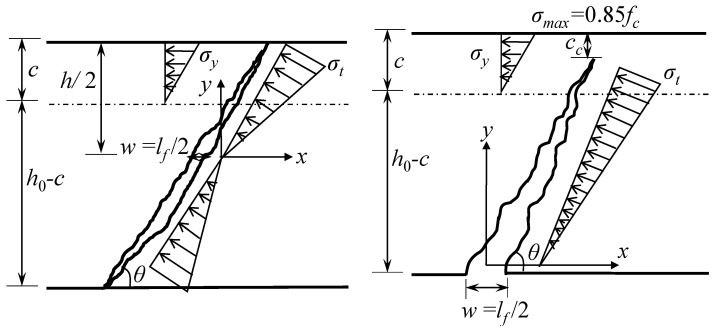
Simplified calculation model of fiber bridging effect.

When shear compression failure occurs in the compression zone due to the ultra-high compressive strength of UHPC, there is rarely the phenomenon of UHPC collapse. For convenience, the compressive stress in the compression zone is assumed to be approximately linear, with a maximum principal stress ($\sigma_{max}=0.85f_{c}$), which is shown in Figure 14. It can be written as:

(13) $$\sigma_{y}=\frac{0.85f_{c}}{c}y$$

Finally, the shear contribution provided by the compressive strength of UHPC in the compression zone can be expressed as:

(14) $$\begin{array}{cc} V_{c} & =b\int_{0}^{c}\sqrt{f_{c}^{2}-f_{c}\sigma_{y}}dy\\ & =b\int_{0}^{c}\sqrt{f_{c}^{2}\left(1-0.85\frac{y}{c}\right)}dy\\ & =0.74bcf_{c} \end{array}$$

#### 3.1.2. Shear Contribution of Fiber in the Tension Zone

The UHPC tensile stress can be approximated to be distributed symmetrically along the longitudinal axis of the beam when the compression zone exhibits the diagonal tensile failure (model 1). The distribution of tensile stress can be simplified as a triangular linear distribution, as shown in Figure 14. The shear contribution provided by the crack-bridging effect of fibers in the compression and tension zones of the inclined section is:

(15) $$\begin{array}{l} V_{f}=V_{fc}+V_{ft}\\ \;=2\int_{0}^{\frac{h_{0}}{2}}\frac{\sigma_{t}yb\cot\theta }{\left(h-c_{c}\right)}dy\\ \;=\frac{\sigma_{t}bh_{0}\cot\theta }{2} \end{array}$$

where θ is the main crack angle, $\sigma_{t}$ is the micro tensile strength of UHPC, and $h_{0}$ is the effective height and equal to 0.9 h if there is no data available.

When the compression zone exhibits the shear compression failure (model 2), the tensile stress can still be simplified as a triangular linear distribution. The contribution of crack-bridging effect of fibers in compression and tension zone can be expressed as:

(16) $$\begin{array}{l} V_{f}=V_{fc}+V_{ft}\\ \;\;\;=\int_{0}^{h_{0}-c_{c}}\frac{\sigma_{t}yb\cos\theta }{\sin\theta \left(h-c_{c}\right)}dy\\ \;=\frac{\sigma_{t}b(h_{0}-c_{c})\cot\theta }{2} \end{array}$$

where $c_{c}$ is the height of shear zone.

### 3.2. Determination of Key Parameters

#### 3.2.1. The UHPC Tensile Strength Based on Probability

The probability distribution function of fiber length on a cross-section is [51]:

(17) $$f\left(l_{0}\leq l\leq l_{0}+dl\right)=\frac{2}{l_{f}}dl$$

where $l_{f}$ is fiber length and l is fiber length crossing the section.

The fiber angle distribution function on the cross-section is [52]:

(18) $$\;p\left(\theta_{0}\leq \theta \leq \theta_{0}+d\theta ,\;\varphi_{0}\leq \varphi \leq \varphi_{0}+d\varphi \right)=\varphi \left(\theta ,\varphi \right)\sin\theta d\theta d\varphi$$

where θ and φ are the fiber angles of the X-Y and X-Z plane, respectively.

**Figure 15:**
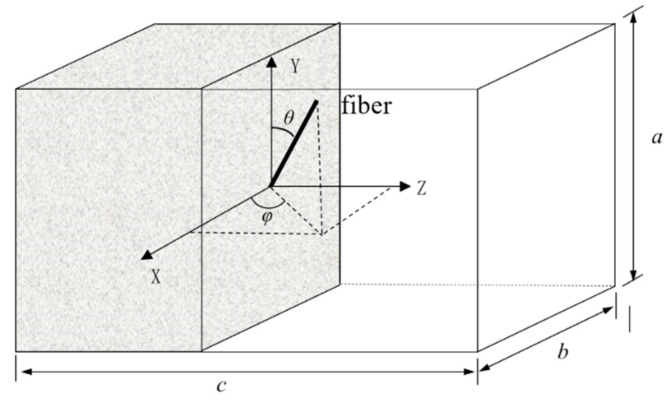
The single-fiber pull-out model.

Since fibers act only in tension on the *Z*-axis (Figure 15), the angle distribution function can be written as follows:

(19) $$p\left(\theta_{0}\leq \theta \leq \theta_{0}+d\theta \right)=\sin\theta d\theta$$

Based on test results, steel fibers typically have a tensile strength greater than 2000 MPa and are usually pulled out from the matrix rather than fracturing when they experience tension failure. If a single steel fiber crosses through a crack parallel to the load, the force required to pull it out from the matrix can be expressed as:

(20) $$\sigma_{f}=2\tau_{max}l_{f}/d_{f}$$

where $\tau_{max}$ is the fiber-matrix bond strength, $l_{f}$ is fiber length, and $d_{f}$ is fiber diameter.

According to the existing research results [53,54], a scratch effect works when a fiber is pulled out from the matrix, and the pull-out force of a fiber can be expressed as:

(21) $$\sigma_{f}=\frac{2{l_{f}\tau }_{max}\left(\theta \right)}{d_{f}}$$

where $\tau_{max}\left(\theta \right)=e^{f\theta }{\left(cos\theta \right)}^{k}\tau_{max,0}$, f=1.2, and k=1  [55].

To calculate the micro tensile strength, a cuboid with a dimension a×b×c along its axis was selected as its element body (see Figure 15). Its volume can be calculated as:

(22) V=abc

In the cuboid, the fiber volume fraction can be expressed as:

(23) $$\rho_{f}=\frac{NA_{f}L}{V}$$

where $\rho_{f}$ is the fiber volume fraction, N is total fiber quantity, $A_{f}$ is the cross-sectional area of single fiber, L is the length of single fiber, and V is the volume of the cuboid.

The action length of a fiber along the *Z*-axis of the cuboid cross-section ($L_{p}$) can be expressed as:

(24) $$L_{p}=L\cos\theta$$

From lengths L to L+dL and angles θ to θ+dθ, the number of fibers crossing any cross-section of the cuboid ($N_{c}$) can be expressed as:

(25) $$N_{c}=\frac{N_{i}L_{p}}{c}$$

where $N_{i}=Nf\left(l\right)P\left(\theta \right)dld\theta$, $L_{p}$ is the action length of the along the *Z*-axis of the cross-section of cuboid, and c is the length of the cuboid.

Therefore, the tensile strength provided by the fiber bridging effect on the cross-section can be expressed as:

(26) $$\begin{array}{l} \sigma_{t}=\left(1/A_{c}\right)\sum_{\theta =0}^{\theta =\frac{\pi }{2}}\sum_{L=0}^{L=\frac{l_{f}}{2}}N_{c}\sigma_{f\theta }A_{f\theta }\\ \;=\frac{4l_{e}\rho_{f}}{d_{f}}\int_{0}^{\pi /2}\int_{0}^{l_{f}/2}f\left(l\right)P\left(\theta \right)\tau_{max}\left(\theta \right)dld\theta \end{array}$$

Equation (27) can be obtained by substituting Equations (17) and (19) into Equation (26).

(27) $$\sigma_{t}=\frac{1.37l_{f}\rho_{f}}{d_{f}}\tau_{max}$$

Voo and Foster [2] described that the bond strength of linear fiber and hooked fiber can be expressed as: (1) $\tau_{max,0}=1.0\sqrt{f_{c}}$; (2) $\tau_{max,0}=0.6\sqrt{f_{c}}$, where $f_{c}$ is the compressive strength of UHPC.

Moreover, it should be noted that the fiber spacing weakens the bond strength between fiber and matrix [56]. Therefore, the bond strength between fiber and matrix, taking the influence of fiber spacing into account, can be represented as follows:

(28) $$\tau_{max}=\tau_{max,0}-Ae^{-\frac{s}{t}}$$

where $\tau_{max,0}$ is the initial bond strength between fiber and matrix, A=2.5, t=0.7, and $s=13.8d_{f}\sqrt{\frac{1}{100\rho_{f}}}$ when the fibers are freely distributed in three dimensions.

#### 3.2.2. Height of Compression and Shear Compression Zone

Zheng [57] reported that the height of the shear compression region ($c_{c}$) decreases linearly with an increase in the shear span ratio (λ).

(29) $$\frac{c}{c_{c}}=\lambda$$

Based on the equilibrium condition of the *X*-axis force, the UHPC beam with diagonal tensile failure in the compression zone can be obtained as:

(30) $$0.5f_{c}bc=V_{f}\tan\theta +\rho_{v}bh_{0}\sigma_{v}$$

where $\rho_{v}$ is the longitudinal reinforcement ratio and $\sigma_{v}$ is the tensile stress of the longitudinal reinforcement at the shear failure of the UHPC beam. Considering the longitudinal reinforcement is in the elastic stage [22], here is selected $\sigma_{v}{=0.3f}_{yv}$, in which $f_{yv}$ is the yield strength of longitudinal reinforcement.

Thus, the height compression zone is:

(31) $$c=\frac{\sigma_{t}bh_{0}+0.6\rho_{v}bh_{0}f_{yv}}{f_{c}b+\sigma_{t}b\frac{1}{\lambda }}$$

Based on the equilibrium condition of the *X*-axis force, the UHPC beam with shear compression failure in the compression zone can be obtained as:

(32) $$0.5\cdot 3.7\sigma_{t}bc=V_{f}\tan\theta +\rho_{v}bh_{0}\sigma_{v}$$

Thus, the height of the compression zone is:

(33) $$c=\frac{\sigma_{t}bh_{0}+0.6\rho_{v}bh_{0}f_{yv}}{3.7\sigma_{t}b}$$

### 3.3. The Calculation Formula for the Shear Capacity of UHPC Beams

The shear capacity of the UHPC beams without web reinforcement can be attributed to the shear contribution of the compression zone and the crack-bridging effect of fiber (Equation (34)), namely:

(34) $$V_{u}=V_{c}+V_{f}$$

In addition to the shear contribution from the above two parts, the web reinforcement in the UHPC beams provides additional shear action (Equation (35)). Thus, the shear capacity of UHPC beams with reinforcement is:

(35) $$V_{u}=V_{c}+V_{f}+V_{s}$$

When the curved prestressing tendons were used in the UHPC beams, the shear contribution provided by the prestressed tendons can be calculated according to the following equation.

(36) $$V_{pd}=f_{pd}A_{p}\sin\delta$$

where $f_{pd}$ is the design strength of prestressed tendon, $A_{p}$ is the cross-sectional area of pre-stressed tendon, and δ is the angle of prestressing tendons.

Therefore, when the compression zone shows tensile failure, the shear capacity of UHPC beams is calculated as:

(37) $$\begin{array}{c} V_{c}=\frac{0.685bcl_{f}\rho_{f}}{d_{f}}\left(k\sqrt{f_{c}}-Ae^{-\frac{s}{t}}\right)\\ V_{f}=\frac{0.685kbh_{0}l_{f}\rho_{f}\cot\theta }{2d_{f}}\left(k\sqrt{f_{c}}-Ae^{-\frac{s}{t}}\right)\\ V_{s}=\frac{A_{s}}{s}h_{0}f_{sy} \end{array}$$

When the compression zone exhibits shear compression failure, the shear capacity of UHPC beams is calculated as:

(38) $$\begin{array}{l} \;\;\;\;\;V_{c}=0.74f_{c}b_{c}\\ \;V_{f}=\frac{0.685l_{f}\rho_{f}b(h_{0}-c_{c})\cot\theta }{2d_{f}}(k\sqrt{f_{c}}-Ae^{-\frac{s}{t}})\\ \;V_{s}=\frac{A_{s}}{s}h_{0}f_{sy} \end{array}$$

where $f_{sy}$ is the yield strength of the stirrup, k is the coefficient of fiber type, and k=0.6 and k=1.0 for linear fiber and hooked fiber, respectively.

### 3.4. Calculation Methods for the Shear Capacity of UHPC Beams in Design

So far, the parts of the UHPC design codes considering the shear capacity calculation of UHPC beams are as follows: a national addition to the Eurocode 2-Design of concrete structures: specific rules for ultra-high performance fiber-reinforced concrete(UHPFRC) [58] and a recommendation: ultra-high performance fiber reinforced cement-based composites (UHPFRC) construction material, dimensioning, and application [59]. The shear capacity calculation formulas in the above design codes are derivative by superposing the shear contribution of the UHPC, fiber, and stirrup. UHPC is typically treated as a homogeneous material in these formulas.

In NF P 18-710 [58], the shear capacity of UHPC structures provided by the matrix ($V_{Rd,\;c}$), the shear capacity provided by the stirrup ($V_{Rd,\;s}$), and the shear capacity provided by the fiber ($V_{Rd,\;f}$) were superposed as follows:

(39) $$\begin{array}{l} \;\;\;\;\;\;V_{u}=V_{Rd,c}+V_{Rd,s}+V_{Rd,f}\\ \;\;\;\;\;\;V_{Rd,c}=\frac{0.21}{\gamma_{cf}\gamma_{E}}k_{p}\sqrt{f_{c}}bh_{0}\\ \;\;\;\;\;\;V_{Rd,s}=\frac{A_{s}}{s}zf_{ys}\cot\theta \\ \;\;\;\;\;\;V_{Rd,f}=\frac{A_{fv}}{\tan\theta }\frac{1}{K\gamma c_{f}}\frac{1}{w_{lim}}\int_{0}^{w_{lim}}\sigma_{f}(w)dw \end{array}$$

where $k_{p}$ is the coefficient considering prestress effect, $\gamma_{cf}\gamma_{E}$ is the factor of safety, z is internal arm height and can be valued 0.9d, θ is the angle between the principal compressive stress line and beam axis, $A_{fv}$ is the fiber limited calculation area, $A_{fv}=bz$, K is the directivity coefficient, $w_{lim}$ is maximum crack width, $w_{lim}=0.3\;mm$, and $\sigma_{f}(w)$ is tensile stress corresponding to crack width, w.

In SIA 2052-2016 [59], the fiber bridging effect is coupled with the shear capacity provided by the matrix ($V_{Rd,U}$) and the shear capacity provided by web reinforcement ($V_{Rd,s}$). The contribution of both is added.

(40) $$\begin{array}{l} V_{u}=V_{Rd,U}+V_{Rd,s}\\ V_{Rd,U}=\frac{bz\cdot 0.5\left(f_{Uted}+f_{Utud}\right)}{\tan\alpha }\\ V_{Rd,s}=\frac{A_{s}}{s}zf_{ys}\left(\cot\alpha +\cot\beta \right)\sin\alpha \end{array}$$

where $f_{Uted}$ is the design value of the elastic tensile strength of UHPC, $f_{Utud}$ is the ultimate tensile strength of UHPC, α is stress field inclination, which is approximately equal to the angle between the crack development direction and beam axis, ranging from 30° to 40°, and β is the inclination angle of web reinforcement or longitudinal reinforcement.

## 4. Evaluation of the Shear Capacity Calculation Formula

**Figure 16:**
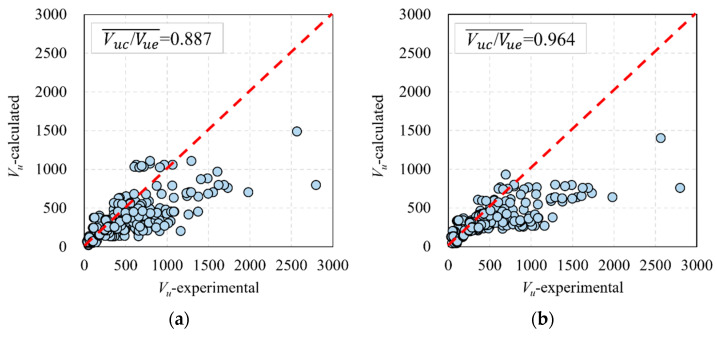

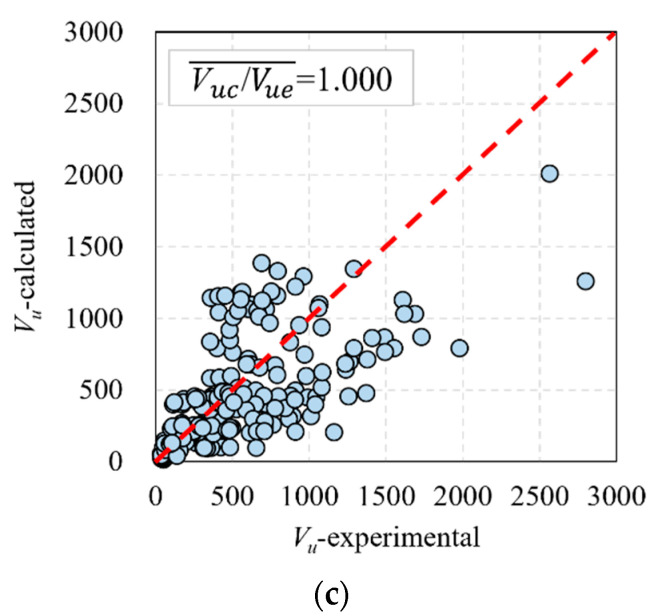
Comparison of formulas proposed in this paper and design codes: (**a**) NF P 18-710 [56]; (**b**) SIA 2052–2016 [57]; and (**c**) calculation of the proposed formula.

The calculation results of these formulas, which were proposed in the above design codes and in this study, are shown in Figure 16. It can be found that the shear capacity calculation formula proposed in this paper accurately predicts the shear capacity of UHPC beams, and the ratio of calculation to test results is 1.00. The ratio of the calculation results of NF P 18-710 and SIA 2052-2016 to test results are 0.887 and 0.964, respectively. It can be concluded that the proposed calculation formula in NF P 18-710, SIA 2052-2016, and this study can all accurately predict the shear-bearing capacity of UHPC beams, whereas the formula proposed in this study gives a higher precision.

## 5. Summary and Conclusions

A UHPC beam shear test database containing 247 samples was analyzed. Then, a novel shear calculation formula for UHPC beams with and without web reinforcement was proposed. The proposed formula fully considers the shear contribution of the fiber bridging effect and UHPC compressive strength, and the following conclusions can be drawn:

(i) The analysis of the shear test database of 247 UHPC beams revealed that the failure of reinforced and prestressed UHPC beams can be categorized into three main types: inclined compression failure, inclined tension failure, and inclined shear compression failure. Specifically, the UHPC beams, in which the shear span ratio is small and has no web reinforcement, tend to exhibit characteristics of diagonal tension failure. This result indicates that, although the fiber bridging effect enhances the shear ductility, its enhancement effect on shear ductility is weaker than that of web reinforcement.
(ii) The nominal shear strength of UHPC beams decreases with an increase in the shear span ratio and increases with an increase in the fiber volume fraction. An increase in fiber volume significantly improves the shear bearing capacity and post-cracking shear behavior of UHPC beams when the fiber volume fraction exists in a certain range.
(iii) The ratio of cracking load to ultimate load of UHPC beams ranges from 0.2 to 0.6. This result indicates that the shear cracking of the UHPC beam generates a low load level. Thus, the shear cracking load level of UHPC beams at serviceability limit states (SLS) should be taken into account in designs to avoid the existence of shear cracks at SLS.
(iv) The failure in the compression zone of UHPC beams can be divided into diagonal tension failure and shear compression failure. Based on the failure mechanism of the compression zone and considering the contribution of fiber micro tensile strength, a formula for calculating the shear bearing capacity of UHPC beams with or without web reinforcement was proposed. Verified by experimental data, the formula accurately predicts the shear-bearing capacity of UHPC beams with an average ratio of 1.0 when compared to test results from the shear database.
(v) It should be noted that the shear-bearing capacity of UHPC beams was greatly affected by the characteristics of UHPC. The same type of UHPC has the same tensile strength but exhibits strain hardening and strain softening constitutive relations, resulting in different shear-bearing capacities. Thus, the calculation of shear-bearing capacity needs to be further related to the constitutive relationship of UHPC.

## Data Availability

Some or all data, models, or code generated or used in this study are available from the corresponding author by request.

## Nomenclature

$A_{f}$	cross-sectional area of single fiber
$A_{fv}$	Fiber-limited calculation area
$A_{p}$	cross-sectional area of the prestressed tendon
a/d	shear span ratio
b	web thickness
c	compression zone height
$c_{c}$	shear zone height
$d_{f}$	fiber diameter
$f_{c}$	compressive strength of UHPC
$f_{dt}$	direct tensile strength of UHPC
$f_{pd}$	design strength of the prestressed tendon
$f_{sy}$	yield strength of the stirrup
$f_{t}$	tensive strength of UHPC
$f_{Uted}$	design value of elastic tensile strength of UHPC
$f_{Utud}$	ultimate tensile strength of UHPC
$f_{yv}$	yield strength of longitudinal reinforcement
h	beam depth
$h_{0}$	effective height
K	a directivity coefficient
k	a coefficient of fiber type
$k_{p}$	a coefficient considering the prestress effect
L	single fiber length
$L_{p}$	action length of a fiber along the z-axis of the cuboid cross-section
l	fiber length crossing the section
$l_{f}$	fiber length
N	total fiber quantity
$N_{c}$	number of fibers crossing any cross-section of cuboid
$N_{i}$	fiber distribution function
V	cuboid volume
$V_{u}$	ultimate load
$V_{cr}$	cracking load
$V_{c}$	shear contribution provided by the UHPC’s compressive strength
$V_{fc}+V_{ft}$	shear contribution provided by the fiber bridging effect
$V_{s}$	shear contribution provided by the web reinforcement tensile strength
$V_{Rd,\;c}$, $V_{Rd,U}$	shear capacity provided by the matrix
$V_{Rd,\;s}$	shear capacity provided by stirrup or web reinforcement
$V_{Rd,\;f}$	shear capacity provided by the fiber
$V_{uc}$	calculated shear capacity
$V_{ue}$	experiment shear capacity
$v_{u}$	nominal shear capacity
$w_{lim}$	maximum crack width
y	height coordinate
z	internal arm height
$\sigma_{1}$, $\sigma_{2}$	principal stress
$\sigma_{f}(w)$	tensile stress corresponding to crack width w
$\sigma_{max}$	maximum principal stress
$\sigma_{t}$	micro tensile strength of UHPC
$\sigma_{v}$	tensile stress of the longitudinal reinforcement at the shear failure
$\sigma_{y}$	normal stress along beam height
$\tau_{0}$	cohesion
$\tau_{max}$	fiber-matrix bond strength
$\tau_{max,0}$	fiber-matrix initial bond strength
$\tau_{xy}$	shear stress
$\left|\tau \right|$	ultimate shearing strength
$\rho_{f}$	fiber volume fraction
$\rho_{s}$	web reinforcement ratio
$\rho_{v}$	longitudinal reinforcement ratio
$\gamma_{cf}\gamma_{E}$	factors of safety
α	stress field inclination
β	inclination angle of web reinforcement or longitudinal reinforcement
δ	prestressing tendons angle
θ	major crack angle
φ	fiber angle of the vertical plane through the fiber direction
ϕ	friction angle
